# First Detection of Hemotropic *Mycoplasma* sp. in Cetaceans of European Waters

**DOI:** 10.3390/vetsci13101090

**Published:** 2026-10-14

**Authors:** Carlos Sacristán, Alba Pereira, Ignacio Vargas-Castro, José Ángel Barasona, José Luis Crespo-Picazo, Daniel García-Párraga, Manena Fayos, Manuel Jesús Varas Cobo, Adrián Wipf Martínez, Teresa Gómez-Tejedor Martín, Aricia Duarte-Benvenuto, Ana de la Torre, Elena Neves, Irene Sacristán, Ana Carolina Ewbank

**Affiliations:** 1 Centro de Investigación en Sanidad Animal (CISA-INIA), Consejo Superior de Investigaciones Científicas (CSIC), 28130 Valdeolmos, Madrid, Spain; torre@inia.csic.es (A.d.l.T.); elena.neves@inia.csic.es (E.N.); irene.sacristan@inia.csic.es (I.S.); 2 Facultad de Ciencias, Universidad Autónoma de Madrid, 28049 Madrid, Spain; alba.pereira@estudiante.uam.es; 3 VISAVET Health Surveillance Centre and Animal Health Department, Veterinary School, Complutense University of Madrid, 28040 Madrid, Spain; ignavarg@ucm.es (I.V.-C.); jbarason@ucm.es (J.Á.B.); 4 Fundación Oceanogràfic de la Comunidad Valenciana, 46005 Valencia, Spain; jlcrespo@oceanografic.org (J.L.C.-P.); dgarcia@oceanografic.org (D.G.-P.); 5 Centro de Recuperación de Fauna Silvestre de Cantabria, 39690 Obregón, Cantabria, Spain; externo.fayos_m@cantabria.es (M.F.); varas_mj@cantabria.es (M.J.V.C.); awipf@tragsa.es (A.W.M.); tere.gtm@gmail.com (T.G.-T.M.); 6 Red de Varamientos de Cantabria (REVARCA), 39690 Obregón, Cantabria, Spain; 7 Faculdade de Medicina Veterinária e Zootecnia, Universidade de São Paulo, São Paulo 05508-270, SP, Brazil; aricia.benvenuto@gmail.com

**Keywords:** bacteria, emerging infectious diseases, Europe, hemoplasma, hemotropic *Mycoplasma*, marine mammals, molecular detection, Mediterranean Sea

## Abstract

*Mycoplasma* spp. are wall-less bacteria classified as hemotropic (hemoplasmas) and non-hemotropic that can cause disease and death in immunosuppressed hosts. While hemoplasmas have been described in cetaceans of the Americas, their presence in cetaceans of Europe remains unknown. In this study, we surveyed hemoplasma in cetaceans stranded in Cantabria (*n* = 20) and the Valencian Community (*n* = 48), Spain. Specifically, we used real-time PCR to test spleen and lung samples from 68 cetaceans of the families Delphinidae (*n* = 60), Phocoenidae (*n* = 4), Kogiidae (*n* = 3), and Ziphiidae (*n* = 1). Overall, 11.8% (8/68) were *Mycoplasma*-positive, all striped dolphins (*Stenella coeruleoalba*) of the Valencian Community. Two nucleotide sequence type (STs) were detected; one was identical to a hemoplasma from a short-beaked common dolphin (*Delphinus delphis*) from Brazil, and the other showed 96.1% nucleotide identity with hemoplasma previously found in Bolivian river dolphins (*Inia boliviensis*), raccoons (*Procyon lotor*), and *Candidatus* Mycoplasma haemolamae of llamas (*Lama glama*) and alpacas (*Vicugna pacos*). Our findings broaden the known geographic range of hemoplasma in cetaceans, reporting for the first time its presence in dolphins in Europe. The health impacts of the detected hemoplasmas in cetaceans are still unknown.

## Simple Summary

*Mycoplasma* spp. are bacteria that can be classified as hemoplasmas, which infect red blood cells, or non-hemotropic *Mycoplasma*, which do not infect blood cells. Hemoplasmas can cause disease and death in animals with compromised immune systems. To date, among cetaceans, hemoplasmas have only been reported in species from the Americas. In this study, we surveyed hemoplasma in 68 cetaceans of Europe, stranded in Spanish regions of Cantabria and the Valencian Community. Hemoplasma DNA was detected in 11.8% (8/68) cetaceans, all striped dolphins of the Valencian Community. Two genetic sequences were detected: one was identical to a hemoplasma previously detected in a common dolphin from Brazil, while the other was more related to hemoplasmas reported in riverine dolphins of Brazil and terrestrial mammals. This study provides the first evidence of hemoplasma in cetaceans in Europe and expands the known geographic distribution of these bacteria. The potential health significance of these findings remains unknown.

## 1. Introduction

Cetaceans are considered sentinels and indicators of aquatic ecosystems health [1,2,3,4]. Their ecological characteristics make them relevant to public health, as some species feed on prey also consumed by people and share coastal ecosystems with them [3]. Additionally, cetacean populations may be negatively impacted by direct and indirect anthropogenic activities, such as fisheries, shipping interaction, chemical, noise and biological pollution, and climate change [5,6,7,8]. These pressures can alter ecosystem health and may influence the emergence and spread of infectious diseases [9]. In cetaceans, emerging pathogens are considered important threats to population health and conservation [10,11]. Some emerging infectious agents affecting cetaceans, such as morbillivirus, herpesvirus, poxvirus, *Brucella ceti* and *Toxoplasma gondii*, have been intensively studied in recent years [10,12,13,14,15,16,17], and are increasingly reported worldwide. In contrast, other emerging infectious agents have only recently been described (e.g., circovirus, pegivirus, polyomavirus, rhabdovirus, *Mycoplasma* spp.) [18,19,20,21,22].

Among the bacterial pathogens of increasing interest are *Mycoplasma* spp. (class Mollicutes)—pleomorphic, small (between 300 and 800 nm in diameter), wall-less bacteria that include hemotropic species (hemoplasmas), as well as species primarily associated with epithelial and mucosal surfaces [23]. These bacteria present extremely reduced genomes and lack many of the metabolic pathways required for independent survival, making them highly dependent on their hosts for essential nutrients and cellular functions [23]. Hemoplasma infections are able to cause disease (e.g., severe anemia) or even death, especially in immunosuppressed or immature individuals, although many hosts develop subclinical infections, and the pathogenicity of some hemoplasma species remains unclear [19,24]. Hemoplasmas are capable of infecting a wide variety of mammals, including domestic species, wildlife, and humans [25]; nevertheless, most of the available knowledge on hemoplasma pathogenicity derives from studies in domestic animals [25]. For instance, there are differences in virulence depending on the hemoplasma species, as observed in *Mycoplasma haemofelis*, considered the most virulent feline hemoplasma, whereas *Mycoplasma ovis* causes disease and significant economic losses in sheep [26,27]. In humans, hemoplasma infections are rare; however, cases of hemolytic anemia associated with *Candidatus* Mycoplasma haemohominis have been documented [25]. In wildlife, the clinical significance of hemoplasma detection remains difficult to determine, particularly when molecular detection is not accompanied by evidence of tissue lesions or other indicators of disease.

Hemoplasmas have been described in two groups of marine mammals, pinnipeds and cetaceans, but have not been detected in sirenians [19,28]. Specifically, among pinnipeds, hemoplasmas have been reported in California sea lions (*Zalophus californianus*) of the US, and in South American fur seals (*Arctocephalus australis*), subantarctic fur seals (*Arctocephalus tropicalis*), and Antarctic fur seals (*Arctocephalus gazella*) of Brazil [19,29,30]. In cetaceans, fully aquatic mammals, hemoplasma sequences have been detected in riverine dolphins, including the Amazon river dolphin (*Inia geoffrensis*), the Bolivian river dolphin (*I. boliviensis*), and the Franciscana dolphin (*Pontoporia blainvillei*), as well as in marine cetaceans, including the Guiana dolphin (*Sotalia guianensis*), pygmy sperm whale (*Kogia breviceps*), dwarf sperm whale (*Kogia sima*), pygmy killer whale (*Feresa attenuata*), common dolphin (*Delphinus delphis*), killer whale (*Orcinus orca*), and rough-toothed dolphin (*Steno bredanensis*) from Brazil [19] and, more recently, in a melon-headed whale (*Peponocephala electra*) from Mexico [31]. Genetic analyses suggest the possible existence of cetacean-specific hemoplasma lineages, as proposed by Duarte-Benvenuto et al. [28]. Nevertheless, the available information on their host range, transmission routes, pathogenicity, and potential zoonotic implications remain very limited in marine mammals [19,28,31]. Such knowledge gap is particularly significant in cetaceans, where hemoplasma occurrence and transmission may be influenced by host species, geographical distribution, ecological characteristics, exposure to different ecosystems, and potential vectors. Although *Mycoplasma* spp. have previously been reported in stranded cetaceans in Europe, specifically in Italy, through bacterial culture [32], this approach does not detect hemoplasmas, which are non-cultivable by conventional bacteriological methods. Therefore, to date, the presence of hemoplasma in cetaceans of Europe has not yet been documented.

We hypothesize that, given the description of *Mycoplasma* spp. in cetaceans from other geographical regions, these bacteria could also be present in cetaceans from Europe. The goal of this study was to assess the presence of hemoplasma DNA in stranded cetaceans along the coast of Cantabria and Valencia, Spain.

## 2. Materials and Methods

We analyzed spleen and lung samples from 68 cetaceans stranded along the Mediterranean coast of the Valencian Community (*n* = 48) and the Atlantic coast of Cantabria (*n* = 20), Spain, comprising the families Delphinidae (*n* = 60), Phocoenidae (*n* = 4), Kogiidae (*n* = 3), and Ziphiidae (*n* = 1). Necropsies followed standardized protocols [33,34], and spleen and lung samples were collected. Lung and spleen were selected because they are highly vascularized tissues, and the spleen is a major site of erythrocyte sequestration and catabolism, making both tissues suitable for hemoplasma DNA detection. Samples were stored frozen at −80 °C until analysis.

In the Valencian Community, we analyzed 55 tissue samples (27 spleens, 28 lungs) from 48 cetaceans stranded between 2016 and 2025: 40 striped dolphins (*Stenella coeruleoalba*), 5 common bottlenose dolphins (*Tursiops truncatus*), 2 short-beaked common dolphins (*Delphinus delphis*), and 1 Risso’s dolphin (*Grampus griseus*), comprising 28 males, 19 females and one individual of unknown sex; 10 calves, 11 juveniles, and 27 adults (Table 1).

In Cantabria, we analyzed 37 tissues of 20 cetaceans (20 spleens, 17 lungs) stranded in 2023 (*n* = 19), and 2024 (*n* = 1): 6 short-beaked common dolphins, 5 striped dolphins, 4 harbor porpoises (*Phocoena phocoena*), 3 pygmy sperm whales (*Kogia breviceps*), 1 long-finned pilot whale (*Globicephala melas*), and 1 Cuvier’s beaked whale (*Ziphius cavirostris*), comprising 11 males and 9 females; 12 adults and 8 juveniles (Table 1).

DNA extraction protocols differed between regions due to sample preservation and laboratory workflows. Each sample (20 mg) from Cantabria was mechanically homogenized in 300 µL of phosphate-buffered saline (PBS) and 500 µL of lysis buffer in 2 ml tubes containing 1.4 mm ceramic beads (Fisherbrand, Pittsburgh, PA, USA) using a Precellys 24 homogenizer (Bertin, Berlin, Germany), centrifuged (5000 rpm for 5 min), and collected for nucleic acid purification (700 µL of the supernatants). Nucleic acids were automatically extracted using the IndiMag Pathogen IM48 Cartridge (Indical Bioscience GmbH, Leipzig, Germany) in the IndiMag 48 (Indical Bioscience). Samples from Valencia were homogenized using stainless steel 4.8 mm beads (Next Advance, Averill Park, NY, USA) after being added to PBS at a 1:10 proportion. DNA was extracted using the High Pure Template Preparation Kit (Roche Diagnostics, Mannheim, Germany) or the High Pure Viral Nucleic Acid Kit (Roche Diagnostics). All procedures followed the manufacturers’ instructions.

**Figure 1:**
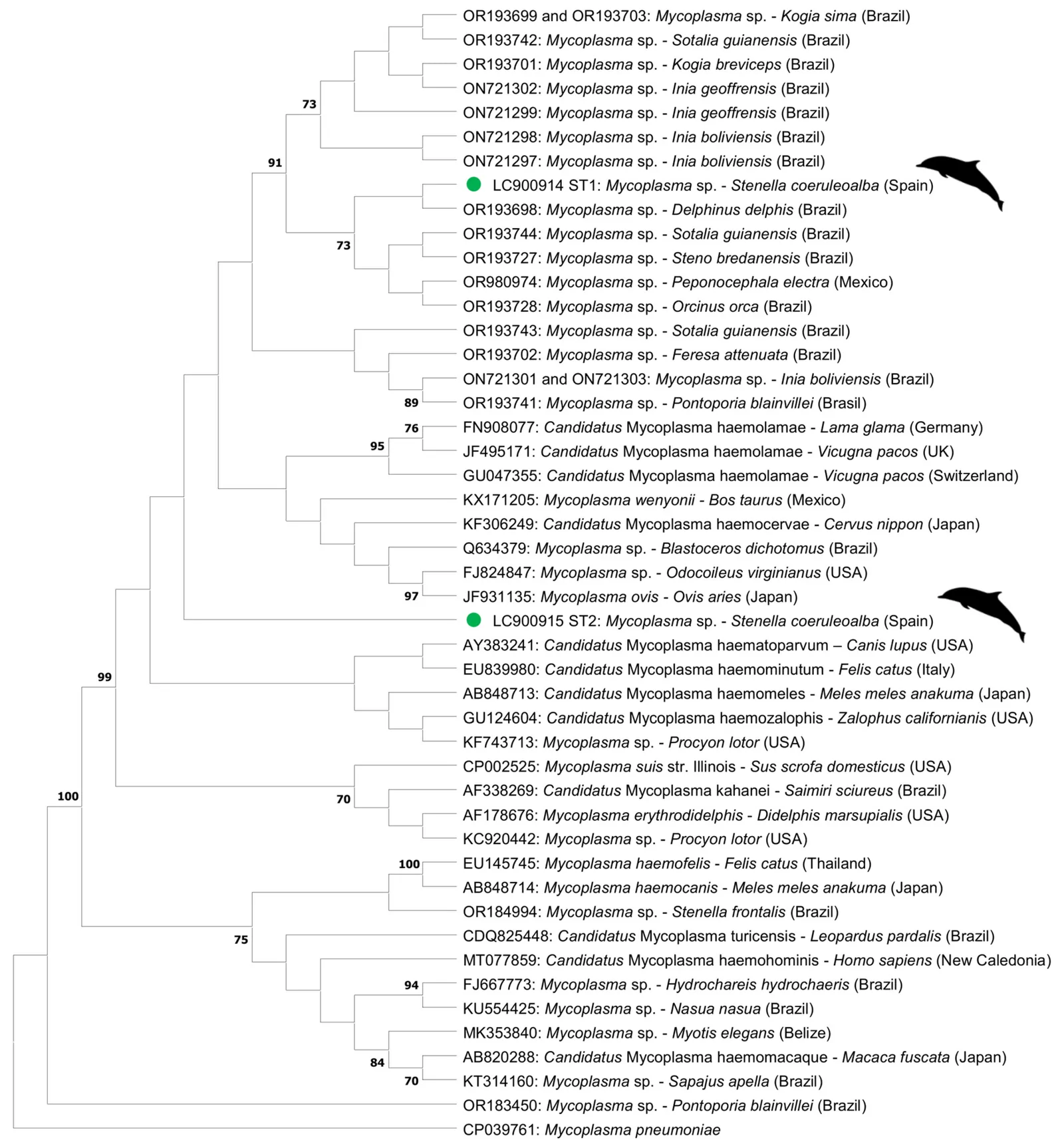
Maximum likelihood phylogenetic tree based on the GTR model with invariant sites and gamma distributed of the nucleotide sequences (i) obtained in this study (green dots and dolphin silhouettes), (ii) the closest from GenBank/ENA/DDBJ database, (iii) other hemoplasma sequences obtained in cetaceans and other mammals, and (iv) an epitheliothropic *Mycoplasma* sp. sequence identified in a cetacean (OR183450). *Mycoplasma pneumoniae* was used as outgroup. The evolutionary model was selected using jModelTest v2.1.10 (My Biosoftware, https://mybiosoftware.com). All bootstrap frequency values < 70 were omitted. The length of the sequences after excluding primers was 332 bp.

Samples were analyzed for *Mycoplasma* spp. DNA using a real-time PCR protocol amplifying a fragment of 16S rRNA gene, with the primers myco16S rRNA-F (5′-ATGTTGCTTAATTCGATAATACACGAAA-3′) and myco16S rRNA-R (5′-ACRGGATTACTAGTGATTCCAACTTCAA-3′) [35,36]. Each 20 µL reaction contained 10 µL of KAPA SYBR Fast Master Mix (2X, KAPA Biosystems, Cape Town, South Africa), 0.4 µL of each primer at an initial concentration of 20 µM, 5.2 µL of nuclease-free water, and 4 µL of DNA template. PCR amplification was performed under the following conditions: initial denaturation at 95 °C for 3 min, followed by 40 cycles of denaturation at 95 °C for 15 s and annealing/extension at 60 °C for 60 s [35,36]. Appropriate positive and negative controls were included in each PCR run. Amplification products were expected to yield fragments ranging from 334 to 391 bp. Amplicons of the expected size were purified using Exo-SAP IT (Thermo Fisher Scientific, Austin, TX, USA) and directly sequenced by Sanger sequencing in both directions. The obtained nucleotide consensus sequence types (STs) were compared with the closest sequences available at GenBank by BLASTn (NCBI) (version 2.17.0) and aligned with Clustal2 for p-distance calculation using Mega 11, after primers exclusion. Mega 11 was used to construct the maximum-likelihood phylogram (Figure 1). The evolutionary model was selected using jModelTest v2.1.10 (My Biosoftware, https://mybiosoftware.com) (accessed on 20 August 2026).

Associations between *Mycoplasma* prevalence and sex, age class, region, and season (warm vs. cold season) were analyzed. The individual animal was considered the unit of analysis, and an animal was classified as positive if either the spleen or lung tissue tested hemoplasma-positive. The response variable (Myco) was coded as binary (0 = negative, 1 = positive).

Due to the low number of positive individuals (*n* = 8), multivariable logistic regression was not performed in order to avoid model overfitting. Instead, associations between *Mycoplasma* detection and each explanatory variable (sex, age class, region and season—cold season: autumn–winter; warm season: spring–summer (based on stranding date)) were evaluated using separate univariable penalized logistic regression models. Firth’s penalized maximum likelihood approach was applied to reduce small-sample bias and to account for potential separation issues arising from sparse data [37]. Odds ratios (OR) and 95% confidence intervals (95% CI) were calculated from the models. Global *p*-values for each explanatory variable were obtained using penalized likelihood ratio tests. Statistical significance was set at *p* < 0.05.

Univariable logistic regression models with Firth’s penalized likelihood were fitted using the logistf package [37] in R version 4.5.2 (R Foundation for Statistical Computing, Vienna, Austria).

## 3. Results

Overall, 11.8% (8/68; 95% CI: 5.2–21.9) of the cetaceans were *Mycoplasma*-positive, all from the Valencian Community (16.6%, 8/48), with 14.3% (4/28) of lung and 14.8% (4/27) of spleen samples testing positive. Among the eight *Mycoplasma*-positive individuals, seven presented either spleen or lung tissue available, and only one individual—which tested positive in the lung—presented both tissues (spleen and lung). All samples from Cantabria (20 spleens, 17 lungs), collected from 20 cetaceans, were *Mycoplasma*-negative.

All eight *Mycoplasma*-positive cases found in this study were adult striped dolphins, representing 17.7% (8/45) of the overall striped dolphins—40 from the Valencian Community and five from Cantabria. The positive striped dolphins comprised 6 males and 2 females of the Valencian Community. In univariable Firth’s penalized logistic regression models, none of the explanatory variables showed strong statistical association in overall tests, although these results should be interpreted cautiously given the small number of positive individuals (*n* = 8, Table 2). Two nucleotide STs were identified: (i) ST-1, detected in four animals and presenting 100% nt identity to a hemoplasma of a short-beaked common dolphin from Brazil (OR193698); and (ii) ST-2, found in other four individuals and presenting the highest nt identity (96.1%) with hemoplasma from Bolivian river dolphins (*Inia boliviensis*) of Brazil (ON721301; ON721303), raccoons (*Procyon lotor*) of the USA (e.g., KC936280, KF743713, KF743717), and *Candidatus* Mycoplasma haemolamae of llamas (*Lama glama*) from Germany (FN908076, FN908077) and alpacas (*Vicugna pacos*) from the UK (JF495171). The retrieved sequences clustered with other hemoplasmas in the phylogram, specifically ST-1 grouped with hemoplasmas of Delphinidae, while ST-2 clustered with hemoplasma from aquatic and terrestrial mammals (Figure 1). Representative STs were submitted to DDBJ under accession numbers LC900914 and LC900915.

## 4. Discussion

Our findings broaden the geographic range of hemoplasma in cetaceans, confirming its presence in dolphins in Europe. This is the second report in striped dolphins, following a previous description in Brazil, despite the low quality of the earlier sequence [19]. The prevalence observed in cetaceans of Spain (11.8%, 8/68) was similar to that reported in Brazilian marine cetaceans (14.8%, 18/130) but was apparently lower than in riverine dolphins of Brazil (64.0%, 32/50) [19,28]. All hemoplasma-positive striped dolphins were from the Mediterranean Sea. The striped dolphin is currently considered the most abundant cetacean species in the Mediterranean Sea, particularly in the western Mediterranean, with approximately 750,000 individuals estimated for the entire Mediterranean Sea [38]. This subpopulation has been classified as “Least Concern” according to the Red List of the International Union for Conservation of Nature (IUCN), although it faces several threats, including overfishing, poor habitat quality, exposure to pathogens, pollution and climate change [39]. Notably, Mediterranean striped dolphins have historically been affected by large-scale epizootics, such as the mass mortality associated with morbillivirus between 1990 and 1992, and exposed to multiple stressors, such as infectious diseases [39]. Understanding the epidemiology of newly described pathogens is particularly important in such populations, especially from a conservation perspective. Although we detected hemoplasma DNA, it does not necessarily imply that the microorganism is associated with clinical disease. Unfortunately, no clinical, hematological, or histopathological information was available for the stranded animals included in this study. Further investigation is warranted to assess hemoplasma relevance to cetacean health surveillance.

Hemoplasma-positive cases were only detected in the Mediterranean coast of Valencian Community, while all the 20 cetaceans tested in the Atlantic coast of Cantabria were hemoplasma-negative. All positive cases were detected in striped dolphins, the most frequently represented species in the Valencian dataset. Our findings in Cantabria should not be interpreted as evidence of absence, especially given the smaller sample size and differences in species composition and sampling periods between the two studied regions. Thus, the observed geographic pattern may partly reflect differences in host composition, sampling and DNA extraction method rather than a true regional restriction of hemoplasma occurrence. Broader sampling involving multiple cetacean species and geographic areas is necessary to determine if the Mediterranean distribution found in this study would be maintained in larger datasets.

We did not detect an association between hemoplasma detection and the explanatory variables evaluated herein; however, the apparent concentration of positive cases in adults and their absence in calves and juveniles warrant further investigation. Due to the low number of positive animals (only eight), the statistical estimates were imprecise, showing wide confidence intervals, and the study had limited power to detect associations. The interpretation of the statistical associations is also limited by the species composition of the sample, as all eight positive animals were striped dolphins, making it difficult to disentangle species-related effects from those of the other variables evaluated. Therefore, these results should be considered exploratory and hypothesis-generating rather than confirmatory. Larger datasets will be needed to determine whether the observed distribution among adults reflects differences in cumulative exposure or susceptibility, or simply the composition of the sampled population.

Based on the phylogram, the ST-1 clustered with other hemoplasma sequences retrieved from delphinids, whereas ST-2 was more divergent and clustered, with 99% bootstrap support, within a clade comprising hemoplasma sequences from cetaceans, other cetartiodactyls and carnivores. Additionally, ST-2 presented low nt identity with known hemoplasma sequences for a well-conserved gene, the 16S rRNA. However, the divergent ST-2 sequence cannot be reliably placed among hemoplasma lineages based on the partial 16S rRNA sequence alone. Additional genetic markers or whole-genome data would provide greater phylogenetic resolution and help clarify the relationship of ST-2 with other hemoplasma lineages. The hemoplasma ST-1 was identical to a sequence previously described in a short-beaked common dolphin of Brazil [19]. This finding is particularly interesting given the geographical distance between the two regions and may reflect the occurrence of a hemoplasma lineage in geographically distant cetacean populations, as proposed for *Helicobacter cetorum* [40]. Possible explanations include shared evolutionary origin, historical connectivity, or the presence of a widely distributed hemoplasma, but the present dataset does not allow these alternative explanations to be distinguished. Of note, the striped dolphin has a broad geographic distribution, inhabiting warm and temperate waters throughout the Atlantic, Pacific, and Indian Oceans, as well as the Mediterranean Sea [41]. However, the broad geographic distribution of the species should not be interpreted as evidence of regular transoceanic movements of individuals. Genetic studies indicate differentiation between Mediterranean and Atlantic populations, with limited gene flow across the Strait of Gibraltar [42,43]. Nevertheless, the introduction of pathogens from the Atlantic to the Mediterranean cetacean populations has been recorded, specifically the dolphin morbillivirus [44]. Broader sampling across the Atlantic and Mediterranean, together with additional genetic markers, are needed to clarify the origin and distribution of ST-1.

Hemoplasma transmission routes in marine mammals remain unclear. As fully aquatic mammals, cetaceans have limited opportunities for exposure to terrestrial vectors. Nevertheless, direct contact, aggressive interactions, and vertical transmission may represent possible transmission routes, as described in terrestrial species [25].

## 5. Conclusions

Our results provide new insights into the geographical range and genetic diversity of hemoplasmas in cetaceans, despite a limited sample size and study period. The significance of hemoplasma detection in striped dolphins should ultimately be evaluated in the context of the cumulative stressors affecting Mediterranean cetaceans. We recommend the incorporation of hemoplasma screening into standardized cetacean health surveillance, together with molecular, pathological, ecological, and individual health data, to clarify the significance of hemoplasma detection in cetaceans. This integrated approach could improve our understanding of hemoplasma epidemiology in cetaceans, support the early detection of emerging infectious agents, and contribute to cetacean conservation.

## Figures and Tables

**Table 1:** Tissue samples, region, sampling period, species, sex, and age class of cetaceans of Spain tested for *Mycoplasma* spp. M = male, F = female, U = unknown, C = calf, J = juvenile, A = adult.

Region and Sampling Period	Species	N	Tissue Samples	Sex (M/F/U)	Age Class (C/J/A)
Valencian Community (2016–2025)	*Stenella coeruleoalba*	40	47 (24 spleens, 23 lungs)	23/16/1	9/7/24
	*Tursiops truncatus*	5	5 (3 spleens, 2 lungs)	2/3/0	0/3/2
	*Delphinus delphis*	2	2 (0 spleen, 2 lungs)	2/0/0	1/0/1
	*Grampus griseus*	1	1 (0 spleen, 1 lung)	1/0/0	0/1/0
Cantabria (2023–2024)	*Delphinus delphis*	6	12 (6 spleens, 6 lungs)	6/0/0	0/2/4
	*Stenella coeruleoalba*	5	9 (5 spleens, 4 lungs)	1/4/0	0/2/3
	*Phocoena phocoena*	4	7 (4 spleens, 3 lungs)	1/3/0	0/1/3
	*Kogia breviceps*	3	6 (3 spleens, 3 lungs)	2/1/0	0/2/1
	*Globicephala melas*	1	1 (1 spleen, 0 lung)	0/1/0	0/1/0
	*Ziphius cavirostris*	1	2 (1 spleen, 1 lung)	1/0/0	0/0/1
**Total**		**68**	**92 (47 spleens, 45 lungs)**	**39/28/1**	**10/19/39**

**Table 2:** Prevalence of *Mycoplasma* and results of univariable Firth’s penalized logistic regression models evaluating associations between detection status and explanatory variables (sex, age class, region and season) in stranded cetaceans (*n* = 68). The reference category for each variable is indicated by OR = 1. The *p*-values shown correspond to category-specific comparisons against the reference level, while overall *p*-values were obtained from penalized likelihood ratio tests evaluating each variable as a whole.

Variable	Category	N	Positives and Prevalence (%)	OR (IC 95%)	*p*-Value	Overall *p*-Value
Sex	Female	28	2 (7.1)	reference	—	0.57
	Male	39	6 (15.4)	2.06 (0.48–11.92)	0.343	
	Unknown	1	0 (0)	3.53 (0.02–90.02)	0.512	
Age class	Adult	39	8 (20.5)	reference	—	0.056
	Calf	10	0 (0)	0.18 (0–1.64)	0.149	
	Juvenile	19	0 (0)	0.1 (0–0.84)	0.031	
Region	Cantabria	20	0 (0)	reference	—	0.052
	Valencia	48	8 (16.7)	8.6 (0.99–1132.98)	0.052	
Season	Cold season	48	6 (12.5)	reference	—	0.8
	Warm season	20	2 (10)	0.88 (0.15–3.9)	0.87	

## Data Availability

The original data presented in the study are openly available in GenBank at accession numbers LC900914 (https://www.ncbi.nlm.nih.gov/nuccore/LC900914 accesed on 1 October 2026) and LC900915 (https://www.ncbi.nlm.nih.gov/nuccore/LC900915 accesed on 1 October 2026).
